# Clinical Applications of Astaxanthin in the Treatment of Ocular Diseases: Emerging Insights

**DOI:** 10.3390/md18050239

**Published:** 2020-05-01

**Authors:** Giuseppe Giannaccare, Marco Pellegrini, Carlotta Senni, Federico Bernabei, Vincenzo Scorcia, Arrigo Francesco Giuseppe Cicero

**Affiliations:** 1 Department of Ophthalmology, University Magna Graecia of Catanzaro, 88100 Catanzaro, Italy; giuseppe.giannaccare@gmail.com (G.G.); vscorcia@libero.it (V.S.); 2 Ophthalmology Unit, S.Orsola-Malpighi Hospital, University of Bologna, 40138 Bologna, Italy; marco.pellegrini@hotmail.it (M.P.); c.senni3@gmail.com (C.S.); federico.bernabei89@gmail.com (F.B.); 3 Medical and Surgical Sciences Department, Alma Mater Studiorum University of Bologna, 40138 Bologna, Italy

**Keywords:** astaxanthin, carotenoids, eye, age-related macular degeneration, glaucoma, cataract, dry eye disease, oxidative stress, nutritional supplements

## Abstract

Astaxanthin is a naturally occurring red carotenoid pigment belonging to the family of xanthophylls, and is typically found in marine environments, especially in microalgae and seafood such as salmonids, shrimps and lobsters. Due to its unique molecular structure, astaxanthin features some important biologic properties, mostly represented by strong antioxidant, anti-inflammatory and antiapoptotic activities. A growing body of evidence suggests that astaxanthin is efficacious in the prevention and treatment of several ocular diseases, ranging from the anterior to the posterior pole of the eye. Therefore, the present review aimed at providing a comprehensive evaluation of current clinical applications of astaxanthin in the management of ocular diseases. The efficacy of this carotenoid in the setting of retinal diseases, ocular surface disorders, uveitis, cataract and asthenopia is reported in numerous animal and human studies, which highlight its ability of modulating several metabolic pathways, subsequently restoring the cellular homeostatic balance. To maximize its multitarget therapeutic effects, further long-term clinical trials are warranted in order to define appropriate dosage, route of administration and exact composition of the final product.

## 1. Introduction

**Figure 1:**
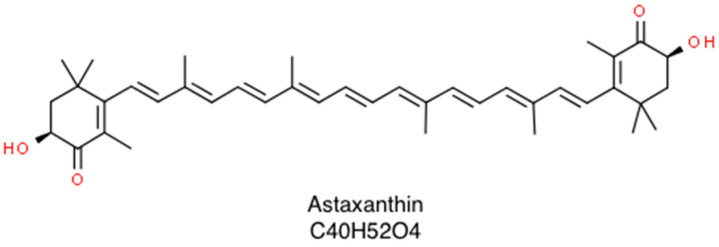
Skeletal structure of astaxanthin that consists of a β,β-carotene-4,4’-dione bearing two hydroxy substituents at positions 3 and 3’.

Astaxanthin (3,3’-dihydroxy-β,β-carotene4,4’-dione; molar mass 596.84 g/mol) is a naturally occurring carotenoid whose structural and functional characteristics make it a promising bioactive compound in the prevention of several human diseases as well as in the maintenance of a good health status [1]. It belongs to the family of xanthophylls (the oxygenated derivatives of carotenoids) and is especially common in marine environments where it looks like a red pigment. This contributes to the pinkish-red color of salmonids, shrimps, lobsters, and crayfish’s flesh [2]. Once biosynthesized by phytoplankton and microalgae, such as *Haematococcus pluvialis*, *Chlorella zofingiensis* and *Xanthophyllomyces dendrorhous*, it accumulates in various aquatic species, which represent the main dietary sources of such valuable nutrient. As a member of xanthophylls, it consists of two terminal rings joined by a polyene chain, plus two asymmetric carbons located at the 3,3′ positions of the β-ionone ring with hydroxyl group (-OH) on either end of the molecule, which provide both lipophilic and hydrophilic properties. Each double polyene bond can exist either as *cis-* or *trans*-geometric isomers. Further, due to the presence of two stereogenic carbon atoms at the 3,3’ positions, three different stereoisomers of astaxanthin, including a pair of enantiomers (3*R*,3’*R*- and 3*S*,3’*S*-astaxanthin) and an optically inactive mesoform (3*R*,3’*S*-astaxanthin) may be found, as shown in Figure 1 [1]. 

Application fields of astaxanthin range from the food-coloring industry, for its natural intense red color brightness, to aquaculture and poultry industries for its natural feed addictive properties [3]. Furthermore, the use of astaxanthin in the medical field is of recent interest, with the purpose of improving health status. Not by chance, it has shown multiple beneficial effects including anticancer, antidiabetic, anti-inflammatory and antioxidant activities, as well as protective actions for skin and nervous and cardiovascular systems [1,4,5]. Currently, 95% of astaxanthin available in world market is produced synthetically using petrochemicals due to cost-efficiency for mass production; recently however, the interest in natural sources of the pigment is increasing substantially [6,7].

Unlike most antioxidants which work in the inner (e.g., vitamin E and β-carotene) or in the outer side of the membrane (e.g., vitamin C), astaxanthin stretches through the bilayer membrane, providing protection against oxidative stress by scavenging reactive oxygen species (ROS) in both the inner and outer layers of the cellular membrane [4,5].

Several recent clinical trials highlight the potential role of astaxanthin in promoting eye health, as suggested by the significant improvement in the outcomes of various ocular diseases including diabetic retinopathy, age-related macular degeneration, glaucoma and cataract. 

The aim of the present paper is to provide a comprehensive review of the current clinical applications of astaxanthin in the treatment of a wide range of ocular diseases. 

## 2. Literature Review: Methods 

Relevant articles published to March 2020 were searched using PubMed, Scopus and Medline databases, as well as through the reference lists of identified publications. Search terms included the following key phrases: astaxanthin; carotenoids; eye; age-related macular degeneration; glaucoma; cataract; dry eye disease; oxidative stress; nutritional supplements. 

## 3. Mechanism of Action

### 3.1. Antioxidant Activity

Oxidative stress results from a disturbance of the delicate balance between cellular pro- and anti-oxidant reactions, and acts as a key mediator in the pathogenesis of several human diseases. Indeed, the excess of oxidative agents may react with proteins, lipids and nucleic acids through a chain reaction with subsequent functional and structural damage. A common carotenoid exerts its antioxidant activity either by disrupting free-radical chain reactions or by reacting with them to produce harmless products, whereas astaxanthin neutralizes single oxygens and scavenges radicals to prevent chain reactions, thus preserving membrane structure [5]. Naguib and coauthors showed that astaxanthin has a higher antioxidant activity compared to other carotenoids such as α-carotene, lycopene, lutein, and β-carotene [8]. Furthermore, another study evaluated the effects of astaxanthin, zeaxanthin, lutein, β-carotene and lycopene on lipid peroxidation [9]. The nonpolar carotenoids, lycopene and β-carotene disordered the membrane bilayer and stimulated membrane lipid peroxidation, whereas astaxanthin preserved membrane structure and exhibited significant antioxidant activity [9,10]. Due to its molecular array, astaxanthin is able to bind to and span the cell membrane, subsequently neutralizing free radicals both in the nonpolar (hydrophobic) and polar (hydrophilic) boundary zones [4,10]. Clinically, the antioxidant efficacy of such a bioactive compound has been confirmed by several studies, which report a significant reduction in the levels of oxidative markers, such as malondialdehyde (MDA) and isoprostane, and increased levels of antioxidant agents such as superoxide dismutase (SOD) [11,12,13]. By recovering the balance between pro-oxidative and antioxidative agents, astaxanthin addresses a major feature of various ocular diseases affecting both anterior and posterior segment of the eye that are driven by a pro-oxidative environment. Light penetrates every layer of the eye, generating a large volume of ROS, and acquired factors such as persistent hyperglycemia, elevated intraocular pressure (IOP) and inflammatory stimuli contribute to accelerating oxidative reactions. As a result, either cell death or cellular dysfunction may occur leading to keratopathies, cataract formation, and retinopathies. As astaxanthin efficiently prevents ROS formation and exerts high scavenging activity towards hydroxyl radicals, treatment with this xanthophyll micronutrient is expected to halt both the onset and the progression of ROS-related diseases, as demonstrated by the reduced death of retinal ganglion cells and increased stability of the ocular surface unit. [14,15,16] 

### 3.2. Anti-Inflammatory Activity

Inflammation and oxidative stress act as components of a double-ended relationship. Inflammatory mediators such as interleukin (IL)-1B, IL-6, IL-8 and TNF-α, which are upregulated in many ocular diseases, increase the expression of ROS, which likewise lead to enhanced secretion of inflammatory cytokines, chemokines, and matrix-remodeling factors, thus altering local environment homeostasis, and finally contributing to the maintenance of a vicious circle of chronic inflammation and oxidative stress. While a prompt inflammatory response is somehow adaptive in the setting of infection or external injury, it may be detrimental, as robust production of inflammatory mediators can lead to cellular damage, pathological neovascularization and subsequent functional impairment. In the setting of inflammatory-related ocular diseases, several effectors of the immune response become activated by endogenous intra- and extra-cellular danger signals inducing an inflammatory cascade. For instance, degenerative changes in retinal pigment epithelial (RPE) cells trigger a vicious circle promoting the development of chronic inflammation and oxidative stress in the retina and choroid. Persistent stress to the ocular surface, including tear hyperosmolarity, quantitative and/or qualitative changes of tears, and ultraviolet exposure also contribute to inflammatory involvement of the entire ocular surface. As a consequence, increased levels of cytokines have been documented in tears and aqueous humor of patients suffering either with corneal or retinal diseases [17,18]. Inflammatory biomarkers may also guide treatment, as therapies addressing inflammation are much more effective than symptomatic therapies alone in halting the vicious circle underlying chronic eye disorders. It has been reported that astaxanthin can suppress H_2_O_2_-induced NF-κB activation by inhibiting intracellular ROS accumulation. Indeed, increasing levels of oxidative agents may augment NF-κB activation by enhancing the rapid degradation of its inhibitor, IκB, thus allowing nuclear translocation of the transcription factor NF-κB and subsequent expression of several inflammatory mediators including iNOS, COX-2, TNF-α, and IL-1β [19]. In addition, as reported by Macedo and coauthors, astaxanthin significantly improves neutrophil phagocytic and microbicidal capacity mostly by reducing deleterious effects of oxidative stress and by increasing intracellular calcium concentration [20]. Indeed, immune cells are particularly sensitive to lipid peroxidation processes due to their high content of polyunsaturated fatty acids in membranes, and generally produce much more oxidative agents because of the important metabolic activity [21]. As a confirmation of such immunoregulatory effect, young healthy females showed enhanced cell-mediated and humoral immune responses following astaxanthin dietary supplementation, whereas reduced bacterial load and gastric inflammation were documented in *Helicobacter pylori*-infected mice after treatment [22,23].

### 3.3. Antiapoptotic Activity

Apoptosis is a form of programmed cell death with a crucial role in mammalian development and tissue homeostasis. Because of this important biologic activity, apoptosis acts as a key pathogenic factor in several age-related human conditions, such as neurodegenerative disorders, ischemic stroke and heart disease [24]. Astaxanthin exhibits either antiapoptotic or proapoptotic effects depending on the pathological condition. Indeed, astaxanthin has been shown to induce cancer cell apoptosis through a mitochondrial-dependent pathway [25]. On the other hand, astaxanthin has been shown to significantly reduce retinal ganglion cells apoptosis that is responsible for the progression of retinal damage in glaucoma and in other optic neuropathies, as well as RPE cells death that causes AMD development [26,27]. In particular, it has been shown to enhance the phosphorylation of BAD, the downregulation of cytochrome c, and the activation of caspases 3 and 9 through the regulation of mitogen-activated protein kinase/p38 (p38 MAPK), as well as the stimulation of the PI3K/AKT survival pathway, which in turn lead to the reduction of mitochondrial-related apoptosis [5,26,28,29]. Thus, the aforementioned mechanisms of cellular death modulation may actively contribute to the amelioration of early brain injury as well as the reduction of retinal ganglion cell loss in a mice model of subarachnoid hemorrhage and diabetic retinopathy, respectively [26,30]. Both human and experimental clinical trials supporting eye health showed that astaxanthin improves the outcomes of various ocular diseases, ranging from the anterior to the posterior segment. Potential clinical applications of such nutrients are discussed below.

## 4. Ocular Applications

**Figure 2:**
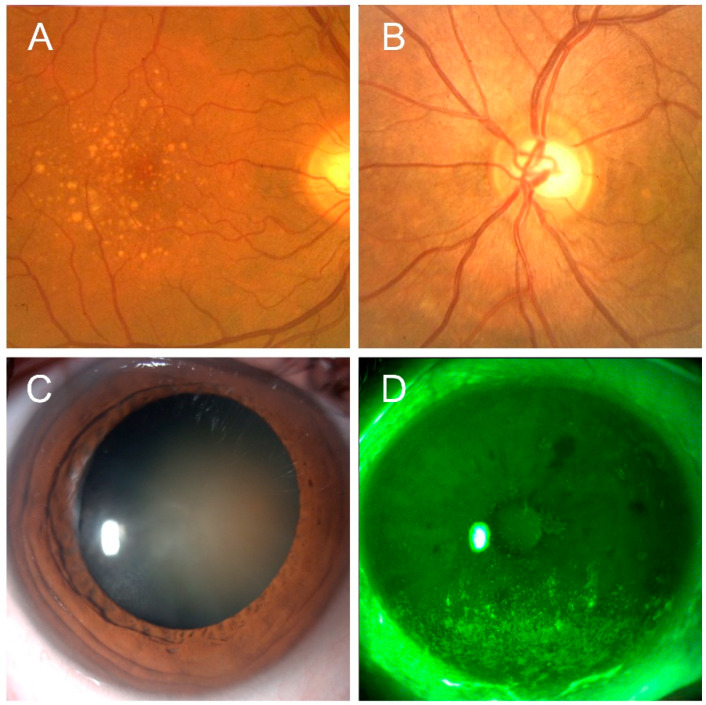
Representative images of ocular conditions that may benefit from the use of astaxanthin: age-related macular degeneration (Panel **A**), glaucoma (Panel **B**), cataract (Panel **C**), keratopathy due to dry eye (Panel **D**) (original pictures from the authors archives).

Preclinical and clinical evidences support the potential use of astaxanthin in the prevention and treatment of a number of ocular diseases shown in Figure 2.

### 4.1. Retinal Diseases

The retina is the innermost, light-sensitive nervous layer of the eye and stands as the most metabolically active tissue in the body, with a constantly high demand for oxygen and an almost continuous exposure to light. Both these conditions cause eye vulnerability to irradiation-initiated oxidative damage and subsequent inflammation. Retinal ganglion cell death is a common feature of many retinal disorders such as glaucoma, optic neuropathies and diabetic retinopathy, whereas RPE cells death plays a key role in AMD pathogenesis. The majority of these conditions are characterized by oxidative stress, ischemia and apoptosis as the main pathogenic factors [16,26,31,32,33,34]. 

Age-related macular degeneration is a leading cause of vision loss among adult individuals in developed countries and occurs due to photoreceptor degeneration in the macula, which is responsible for sharp and high-resolution vision. Macular degeneration gradually leads to vision loss and subsequently significantly affects patients’ quality of life and daily productivity. Photoreceptors are exposed to extensive oxidative stress in the form of light and oxygen, so that the outer 10% of photoreceptor segments are shed daily [35]. Resultant debris are then engulfed and removed by retinal pigment epithelium (RPE), which carries out different functions including light absorption, epithelial transport and secretion as well as immune modulation [36]. Because of RPE dysfunction, local homeostatic balance is disturbed and drusen may be found in the RPE-Bruch’s membrane region as established clinical indicators for early and/or dry AMD, as shown in Figure 2, Panel A. The accumulation of such indigestible materials could induce a mechanical displacement of the outer segments and alter the pathway of nutrient exchange between photoreceptors and choriocapillaris [37]. Previous authors that investigated the protective effect of astaxanthin against light-induced retinal damage have confirmed its beneficial role [31,38,39]. In the study by Otzuka, astaxanthin 100 mg/kg inhibited retinal dysfunction evaluated by electroretinogram (ERG) and outer nuclear layer (ONL) thickness in a mouse model of light-induced retinal damage. Furthermore, it concomitantly decreased cellular oxidative stress as demonstrated by the reduction of 8-hydroxy-deoxyguanosine (8-OHdG) levels [39].

Improvement of ERG pattern was also reported in human studies, as suggested by Parisi and coauthors who conducted a comparative study to evaluate the influence of a short-term carotenoid oral supplementation including astaxanthin on retinal function in the setting of AMD. Twenty-seven patients with nonadvanced AMD were enrolled and randomly divided into two groups: 15 patients had oral supplementation of vitamin C (180 mg), vitamin E (30 mg), zinc (22.5 mg), copper (1 mg), lutein (10 mg), zeaxanthin (1 mg), and astaxanthin (4 mg) daily for 12 months, whereas 12 patients had no dietary supplementation. When compared to placebo group, treated patients showed selective improvement of central retina function, where carotenoids are naturally much more represented as endogenous pigments [40].

In another multicenter, prospective, open-label randomized study, AMD patients treated with a combination of lutein/zeaxanthin and astaxanthin over a two-year period were more likely to report a significant improvement in visual acuity, contrast sensitivity and vision-related functions [41]. Furthermore, astaxanthin, due to the stronger antioxidant activity (about ten times higher compared to that of zeaxanthin and lutein), addresses an additional causative factor of AMD that is choroidal neovascularization (CNV). Indeed, CNV develops with oxidative stress and chronic inflammation adjacent to RPE, Bruch’s membrane and choriocapillaris because of vascular endothelial growth factor (VEGF) upregulation. Subsequently, such new leaky sprouting vessels grow into the retina contributing to the pathogenesis of the formerly known wet AMD by bleeding and leaking fluid, causing the macula to bulge up from its normal flat position and leading to distorted central vision [42]. As a demonstration of its efficacy, astaxanthin treatment leads to a significant suppression of CNV in a mouse model of macular degeneration, in which laser photocoagulation was used to induce growth of pathological neovessels. The molecular mechanisms underlying the suppression of CNV mediated by astaxanthin include the downregulation of various inflammatory mediators including ICAM-1, and MCP-1, macrophage-derived VEGF and IL-6, and endothelial derived-VEGFR [43].

Chronic oxidative stress and inflammation are considered main causes of diabetic retinopathy, which is the most serious sight-threatening complication of diabetes. Retinal vasculature is sensitive to small changes in systemic status and vascular lesions appear early following hyperglycemic damage. Increased flux of polyol pathways, enhanced formation of advanced glycation end products (AGEs), upregulated hexosamine pathway and PKC activation result in the augmentation of oxidative agents, which then induce vascular dysfunction including membrane thickening, pericyte drop out and retinal capillary nonperfusion with subsequent retinal ganglion cell loss [16]. Starting from this assumption, the astaxanthin bioactive compound, which contains several double bonds to scavenge ROS, was shown to exert neuroprotective effects in several experimental models of diabetic retinopathy by reducing oxidative stress, inhibiting NF-κB activity and downregulating the expression of downstream inflammatory mediators, which act as principal stimuli of pathological CNV [16,44,45]. In the study by Yeh and coauthors, 50 female Wistar rats received an injection of streptozotocin (STZ) to induce diabetes and were then randomly selected into four groups that received daily for eight weeks: normal saline; 0.6 mg/kg astaxanthin; 3 mg/kg astaxanthin; 0.5 mg/kg lutein. The group treated with astaxanthin showed the preservation of histological and functional outcomes of diabetic retinopathy, which went together with reduced oxidative stress and inflammation, as suggested by reduced levels of oxidative mediators (8-hydroxy-2’-deoxyguanosine, nitrotyrosine, and acrolein), increased levels of antioxidant enzymes (heme oxygenase-1 and peroxiredoxin) and decreased activity of the transcription factor nuclear factor-kappaB (NF-κB) [16]. In another study by Benlarbi-Ben Khedher, the effect of astaxanthin on aldolase reductase (AR) activity, which is a key enzyme in the polyol pathway responsible for the pathogenesis of diabetic microvascular complications, was assessed both ex vivo and in vivo using a rodent model of type 2 diabetes. Samples were fed with astaxanthin (4.8 mg/kg of body weight) once a day for one week. Results showed a marked reduction of AR activity in vivo and ex vivo following astaxanthin supplementation, thus supporting its role in both the prevention and early treatment of diabetic retinopathy [44]. 

Furthermore, the neuroprotective effect of astaxanthin may be usefully adopted in the management of glaucoma, where elevated IOP leads to lamina cribrosa deformation and subsequent blood flow perturbations, thus causing axon loss and retinal ganglion cells apoptosis with the typical damage of the optic nerve, as shown in Figure 2, Panel B [46]. Current therapy for glaucomatous patients is mostly based on IOP-lowering medications; however, this treatment strategy may not be sufficient to stop the progression of the disease, since various other mechanisms including also oxidative stress may take part in the pathogenesis of glaucomatous damage [47]. It has been suggested that oxidative stress, besides directly damaging the retinal ganglion cell layer, may compromise the trabecular meshwork function, which is the major route for aqueous flow from the anterior chamber of the eye [48]. Overall, these findings support the need for the development of neuroprotective therapies for glaucoma. In this regard, astaxanthin has gained growing interest as a multitarget pharmacological agent against neurodegenerative disorders including Parkinson’s disease, Alzheimer’s disease, brain and spinal cord injuries and neuropathic pain, due to its antioxidative, anti-inflammatory and antiapoptotic properties [2]. Indeed, it inhibits the secretion of ILs, TNF-α, intercellular adhesion molecule (ICAM1) and monocyte chemoattractant protein (MCP-1), and stabilizes free radicals by adding them to its long double-bond chain without disrupting free-radical chain reactions, thus preserving cellular structure. Additionally, astaxanthin acts against apoptosis by blocking p-ERk/ERK, cytochrome C, caspase 3,9 and Bax2/Bcl2 ratio [5].

Suppressive effects of astaxanthin on glaucomatous retinal injury were assessed by Cort and coauthors, who conducted an experimental study on mouse models in which elevated IOP was induced by unilaterally cauterizing episcleral vessels. Samples were then randomly divided into two groups which received olive oil or 5 mg/kg/day astaxanthin for a period of eight weeks. At the end of the experimental period, neuroprotective effect of astaxanthin was determined via electrophysiological measurements of visual evoked potentials (VEP) together with the assessment of retinal apoptosis and oxidative markers expression. When compared to controls, elevated IOP increased retinal protein oxidation, which returned to baseline levels in the group treated with astaxanthin. In addition, enhanced apoptosis was detected in all the groups with elevated IOP in contrast to that one treated with astaxanthin, which showed a significant reduction of apoptotic cells percentage. Furthermore, astaxanthin treatment restored altered VEP parameters, which may be considered as a sensitive and reliable method to evaluate the earliest changes in the visual system occurring in case of elevated IOP [32].

In agreement with the aforementioned neuroprotective effects of such xanthophyll carotenoid, the potential use of astaxanthin in the setting of nonarteritic anterior ischemic optic neuropathy (NAION), which is the most common cause of acute ischemic vision loss in people aged over 50 [49] has recently been suggested. So far indeed, there is no effective therapy for NAION, and the outcome is poor. Recent findings of increased plasma levels of oxidative markers in NAION patients suggest the key role of oxidative processes in the pathogenesis of disturbed small-vessel autoregulation in posterior ciliary circulation, which then leads to vascular insufficiency and subsequent optic nerve head ischemia [50]. In this regard, Lin and coauthors investigated the effect of astaxanthin administration in an experimental rat model of NAION and showed that this carotenoid was able to preserve visual function and reduce apoptosis of retinal ganglion cells after ischemic insult. Interestingly, mice were daily supplemented with astaxanthin (100 mg/kg/day) either before or after inducing oxidative stress in the retina by photodynamic treatment. Regardless of the time of astaxanthin administration, all treated groups achieved neuroprotective effects due to astaxanthin effectiveness in downregulating both oxidative and inflammatory cascades. [33].

### 4.2. Uveitis

Uveitis, which is an umbrella term describing a wide group of inflammatory conditions affecting the middle layer of the eye, is a common cause of vision loss and eye pain. The breakdown of the blood–aqueous barrier in uveitis involves the cellular infiltration, the increase in protein permeability and the upregulation of cytokines and chemokines such as TNF-α, IL-6, MCP-1 and MIP-1 in the aqueous humor and uveal regions [51]. Such inflammatory cascade is invariably associated with increased oxidative stress. Indeed, cytokines and chemokines induce intracellular ROS generation by mitochondrial respiratory chain reaction, arachidonic metabolic reactions and the membrane-bound superoxide-generating enzyme NADPH oxidase. These oxidative products in turn enhance the inflammatory cascade by means of NF-κB activation and together alter cellular and molecular targets, destroying normal tissue homeostasis [51,52]. In a study by Ohgami and coauthors, astaxanthin suppressed the development of experimentally induced uveitis in a dose-dependent fashion. Following uveitis induced by a footpad injection of lipopolysaccharide (LPS), astaxanthin or prednisolone were administered intravenously 30 minutes before, at the same time as, or 30 minutes after LPS treatment. Then, the number of infiltrating cells and protein concentration in the aqueous humor collected 24 hours after LPS treatment was determined. As a result, astaxanthin administration significantly decreased the production of NO, PGE2, and TNF-α by directly blocking NOS enzyme activity [44]. Interestingly, ocular anti-inflammatory effect of 100 mg/kg astaxanthin was comparable to that one of 10-mg/kg prednisolone [53]. Similar results were reported by Suzuki and coauthors, who adopted the same designed model of the study cited above, confirming the anti-inflammatory effectiveness of the microalgae-derived carotenoid, as suggested by reduced cellular infiltration, protein concentration and cytokines levels in the aqueous humor of a uveitis mice model [54].

### 4.3. Cataract

Cataract development causes the opacity of the natural lens of the eye, thus leading to decreased visual acuity. It remains one of the main causes of blindness worldwide and surgery is required to restore vision [55]. Lens proteins normally exist under a soluble phase, and such solubility accounts for transparency. Under certain circumstances, such as ageing, diabetes, steroids use and trauma, proteins leave the soluble phase to form high molecular weight aggregates, which account for the light scattering and opacity of the lens [56]. In such cascade of molecular reactions, oxidative stress seems to play a key role since it causes protein modifications, lipid peroxidation and DNA fragmentation, all processes that are believed to contribute to cataract formation. Starting from this assumption, the strong antioxidative power of astaxanthin could be used to prevent cataract development. To date, evidence of astaxanthin use in the setting of age-related cataract, which represents the most common subtype of cataract encountered in clinical practice, is still missing. Conversely, the beneficial activity of astaxanthin has been recently investigated in the setting of an experimental mouse model of cataract due to hyperglycemia and long-time steroid use. Indeed, in an experimental model of steroid-induced cataract, astaxanthin administration efficiently prevented lens opacification and significantly recovered glutathione levels, suggesting antioxidant activity as the main mechanism involved in cataract prevention [57]. Additionally, a recent study showed that astaxanthin supplementation delayed the development and progression of metabolic cataracts by inhibiting oxidative stress in diabetic mouse models, as demonstrated by a significant reduction in the levels of advanced glycation end products [58].

### 4.4. Ocular Surface

The ocular surface, and in particular the cornea and conjunctiva, is almost constantly exposed to sunlight, which consists of wavelengths including UV light, known as a causative factor of oxidative stress in biological systems. As mentioned above, inflammation and oxidative stress are strongly associated and synergistically act as main pathogenic factors of ocular surface disorders [59].

Dry eye disease, which acts as the best model of an impaired ocular surface system, is characterized by a significant increase of oxidative stress markers and ROS, which are responsible for altering epithelial proliferation and differentiation homeostasis, as documented by decreased proliferation, upward migration and increased apoptotic cells, as shown in Figure 1, Panel D [60].

In particular, decreased tear volume, unstable tear film and excessive tear evaporation lead to the creation of a hyperosmolar environment, which then initiate both inflammatory and oxidative cascades [15,61]. On this basis, several clinical trials have been conducted to assess the efficacy of various nutraceuticals in improving tear film stability and meibomian gland dysfunction in patients diagnosed with dry eye [62,63,64]. About astaxanthin, its efficacy in treating dry eye disease has been assessed in the setting of a combined treatment including multiple nutraceuticals. In particular, a prospective randomized double-blinded study was performed to compare the effects of an antioxidant supplement containing anthocyanosides, astaxanthin, vitamins A, C, E, and several herbal extracts (including Cassiae semen and Ophiopogonis japonicus) with placebo in patients suffering from dry eye disease. The supplementation period lasted 8 weeks and patients were followed up every 4 weeks for 16 weeks. Results showed that oral micronutrients supplementation significantly improved tear production and stability while decreased corneal fluorescein staining and tear ROS levels, thus leading to a significant amelioration in both signs and symptoms [65]. The rationale of the use of astaxanthin derives from its capacity to directly address the vicious cycle of the disease, and in particular the underlying inflammation and oxidative stress.

### 4.5. Asthenopia

Asthenopia, otherwise called eye fatigue, is a common condition occurring on a daily cycle that presents with nonspecific symptoms including discomfort, lacrimation, blurry vision, light sensitivity and in more severe cases, pain. Astaxanthin might relieve eyestrain in people using computer monitors as suggested by Nagaki and coauthors, who conducted a double-blind randomized clinical trial by enrolling visual display terminal workers and prescribed them astaxanthin or placebo. Study outcomes were objectively evaluated by using a proper instrumentation to measure eye muscle endurance. Subjects receiving astaxanthin experienced a significant relief from eyestrain compared to the placebo group [66]. Furthermore, it has been reported that in healthy people over 40 years who received astaxanthin at a dosage of 4 or 12 mg once a day for 28 days, the uncorrected far visual acuity significantly improved, and the accommodation time significantly decreased [67]. In addition, a significant improvement of accommodative ability and pupillary constriction capacity were also achieved in subjects following astaxanthin administration either used alone or included in a multiple dietary supplement. Kono and coauthors conducted a randomized double-blinded study to investigate whether the use of multiple dietary supplement containing lutein, astaxanthin, cyanidin-3-glucoside and docosahexaenoic acid (DHA) would improve accommodative ability of aged and older subjects who complained of eye strain. Near-point accommodation (NPA) and subjective symptoms were evaluated both before and after four weeks’ intake. As a result, the supplemented group reported improvement of both NPA and subjective symptoms, such as “stiff shoulders or neck” and blurred vision. The mechanisms underlying this efficacy may be related to the capacity of astaxanthin of determining relaxing effects on ciliary muscle, increasing blood flow in retinal capillaries and decreasing NF-kB in ciliary body [68,69]. All these activities are of great utility due to the widespread use of compact terminals, such as smartphones and tablets, which overload the accommodative system on a daily basis, thus significantly contributing to the development of eye fatigue [68].

## 5. Discussion

There is a growing body of evidence suggesting the beneficial pleiotropic effects of astaxanthin in the prevention and treatment of several ocular diseases, ranging from the anterior to the posterior pole of the eye. As physiological constituents of human tissues, micronutrients deriving from either food intake or nutraceutical products can act as bioactive compounds and influence both morphology and function of ocular tissues by taking part into several metabolic cellular pathways aiming at preserving a homeostatic balance. A wide spectrum of properties including anti-inflammatory and antioxidant activities as well as metabolism regulation make astaxanthin a suitable multitarget pharmacological agent. Furthermore, this natural carotenoid has the advantage to directly address the main pathogenic factors underlying ocular diseases, such as cumulative oxidative stress and chronic subclinical inflammation. Moreover, astaxanthin showed a good safety profile and no adverse events have been reported in any clinical studies [6,7]. Data from both preclinical and clinical studies suggest the importance of an equilibrate and complete nutrition to support eye health. On this basis, nutraceuticals like astaxanthin may be used not only to fill the nutritional gap (supplement use), but also to treat various pathological conditions by synergistically acting with conventional therapies (therapeutic use). Nevertheless, further information from human intervention studies is required to define duration and modalities of astaxanthin application, since most of available evidence is based upon preclinical animal studies. Although positive outcomes have been reported, results should be interpreted with caution as the translation from animal models to human clinical trials is complex. Considered together, the large safety profile of this carotenoid [70] and its potential usefulness in a number of largely prevalent systemic diseases [71,72] opens up new perspectives of treatment aiming at supporting, preserving and improving eye health, as well as contrasting the natural course of ocular diseases. In order to improve its beneficial effects, studies are needed to improve its bioavailability [73], while more data from randomized controlled studies conducted on clearly defined population groups are desirable to define appropriate dosage and exact composition to be used according to the different clinical indication.
